# Superior Migration of the Humeral Head in Chronic Rotator Cuff Disease: A Narrative Review of Pathomechanisms, Diagnosis, and Treatment

**DOI:** 10.7759/cureus.102470

**Published:** 2026-01-28

**Authors:** Mikołaj Zakryś, Katarzyna Zakryś, Szymon Stupnicki, Mateusz Szot, Aleksandra Oparcik, Jakub Tarczykowski, Natalia Kwaśniewska

**Affiliations:** 1 General Practice, Szpital Wojewódzki w Poznaniu, Poznań, POL; 2 General Practice, Uniwersytecki Szpital Kliniczny w Poznaniu, Poznań, POL; 3 General Practice, Wojewódzki Szpital Wielospecjalistyczny im. Dr. Jana Jonstona w Lesznie, Leszno, POL; 4 General Practice, Centrum Medyczne HCP, Poznań, POL; 5 General Practice, Wojskowy Instytut Medyczny - Państwowy Instytut Badawczy, Warszawa, POL

**Keywords:** acromiohumeral interval, massive rotator cuff tears, reverse shoulder arthroplasty, rotator cuff tears, shoulder biomechanics, superior capsular reconstruction, superior humeral head migration, treatment outcomes

## Abstract

Superior migration of the humeral head (HH) refers to pathological cranial displacement of the HH resulting from chronic rotator cuff (RC) dysfunction and loss of dynamic glenohumeral stabilization. This disturbance is reflected by a reduction of the acromiohumeral interval (AHI), defined as the distance between the inferior border of the acromion and the uppermost part of the HH. Reduced AHI is associated with massive irreparable rotator cuff tears (RCTs), rotator cuff arthropathy (RCA), and inferior surgical outcomes.

This narrative review aims to summarize the pathomechanisms, diagnostic value, and treatment implications of superior HH migration in chronic and massive RCTs. Particular emphasis is placed on the clinical relevance of AHI as a marker of disease severity, fatty infiltration, treatment outcomes, and retear risk. While procedures such as partial repair, superior capsular reconstruction (SCR), and lower trapezius transfer (LTT) may improve AHI and shoulder function in selected patients, reverse shoulder arthroplasty (RSA) remains the most reliable option in advanced disease. Although AHI is a simple, inexpensive, and easily accessible marker, it should not serve as a sole criterion for surgical decision-making. Treatment should be tailored to the patient, depending on age, activity demands, and the severity of glenohumeral joint (GHJ) osteoarthritis (OA).

## Introduction and background

Painful shoulder is one of the leading complaints encountered by orthopedic surgeons, with chronic rotator cuff (RC) disease being its main cause [1]. The chronic form of RC disease differs drastically from the acute form in terms of clinical presentation, treatment, and prognosis. Major risk factors for rotator cuff tears (RCTs) include advanced age, a history of trauma, and heavy labor; RCTs more commonly affect males and the dominant arm [2]. Chronic RC disease can lead to rotator cuff arthropathy (RCA), which seriously impairs shoulder biomechanics and leads to joint degeneration. RCA involves three critical features: RC impairment, degenerative changes to the shoulder, and superior migration of the humeral head (HH) [3,4]. Among other reactive changes, elevation of the HH creates impingement on the coracoacromial arch, altering its shape. It is described in the literature as acetabularization, as well as osteophyte formation on the inferior aspect of the acromion [5].

Superior migration of the HH, reflected by the acromiohumeral interval (AHI), is a known feature of RC pathology and shoulder kinematics disruption. It is defined as a reduction of the distance between the inferior border of the acromion and the uppermost part of the HH. In the literature, this distance is referred to as AHI [6] and has many synonyms, such as acromiohumeral distance (AHD), acromiohumeral space, subacromial space height, and subacromial distance. However, AHI and AHD are the most common in the literature. Many papers suggest that an AHI smaller than 6-7 mm can strongly suggest important clinical and radiological RCTs [7,8]. Furthermore, AHI can aid in the decision-making process and optimize treatment recommendations in RCTs. Superior HH migration was classified into five grades according to Hamada et al., with a modification by Walch et al. [9], who further divided grade 4 into grades 4A and 4B. Grade 1 involves shoulders with AHI >6 mm, grade 2 is characterized by AHI ≤5 mm, and grade 3 comprises AHI ≤5 mm and acromion acetabulization. Grade 4 was subdivided into grade 4A (GHJ narrowing but no acromion acetabulization) and grade 4B (GHJ narrowing and acromion acetabulization). Finally, Hamada grade 5 is defined by HH collapse (Table 1) [10]. 

**Table 1:** Hamada classification of the superior humeral head migration with a modification by Walch et al. Source: [9,10] AHI, acromiohumeral interval

Grade	AHI	Acromial acetabulization	Glenohumeral joint	Key features
1	>6 mm	Absent	Normal	Normal joint
2	≤5 mm	Absent	Normal	AHI narrowing
3	≤5 mm	Present	Normal	AHI narrowing and acromial acetabulization
4A	<7 mm	Absent	Narrowing	Glenohumeral joint narrowing without acetabulization
4B	≤5 mm	Present	Narrowing	Glenohumeral joint narrowing with acetabulization
5	Variable	Present	Narrowing/Collapse	Humeral head collapse

This review aims to investigate and highlight the role of the superior migration of the HH as a clinical marker of chronic RCTs, with particular emphasis on pathomechanisms, diagnosis, and treatment. Although the topic of elevation of the HH is widely known and AHI is commonly used, there is an incoherence in research.

## Review

Methodology

This study is a narrative review of the recent literature on the pathomechanisms of the superior migration of the HH and its role in the diagnosis and treatment of chronic RCTs. The PubMed database was searched in November 2025. Peer-reviewed articles in English, published from 2015 onward, were included in this review. Studies that did not refer to superior migration and its clinical relevance were excluded. We focused on systematic reviews and meta-analyses, as they represent the highest level of evidence. However, older studies or studies with a lower hierarchy were also included to provide historical context and when newer sources or studies of a higher hierarchy were not available. As this is a narrative review, no new statistical analyses were performed. All statistical data and quantitative outcomes presented are based exclusively on the results reported in the included primary studies and available meta-analyses.

Pathomechanisms of the HH superior migration

The glenohumeral joint (GHJ) has an unstable nature. It has the greatest range of movement among all joints, yet the HH is not enclosed in a bony acetabulum, as in the hip. The stability of the HH in the glenoid is insufficient, as the glenoid comprises only a quarter of the HH articular surface. Therefore, stability is provided mainly by soft tissue structures, such as the labrum, glenohumeral ligaments, and the RC. The RC acts as a dynamic stabilizer of the HH in the glenoid fossa. It helps keep it centrally directed by compressing it to the glenoid. This phenomenon was described as concavity compression. When the RC function is chronically impaired, shoulder biomechanics become imbalanced, leading to degenerative changes and superior migration of the HH [1,11-13]. Concavity compression and its relevance were clearly explained in a study by Lipitt et al. When a patient is relaxed and RC muscles remain dormant, the examiner can easily translate the HH anteriorly and posteriorly along the glenoid fossa. However, when the subject activates the RC muscles, this movement becomes impossible. Of course, one must not forget the role of the labrum in this mechanism, but this simple demonstration shows how the RC dynamically stabilizes the HH [14].

The synergic influence of the supraspinatus (SSP), infraspinatus (ISP), subscapularis (SSC), and deltoid acts as GHJ stabilizers through compression of the HH to the glenoid fossa. SSP and the deltoid provide stability in the coronal plane, whereas the SSC and ISP support axial stability [11]. Loss of function in the RC muscles, especially through RCTs, causes a reduction in the concavity compression force, and HH stability in the glenoid fossa is impaired. It is important that the forces stabilizing the HH are evenly distributed and act as so-called force couples; otherwise, this imbalance leads to superior migration of the HH through dominance of the deltoid’s upward force [1,11,13,15-17]. Scientific evidence supporting this pathomechanism is strong. In their cadaveric study involving eight shoulders, Mihata et al. demonstrated that a tear of the SSP significantly contributed to HH elevation [15]. Some studies suggest the clinical relevance of the teres minor in stabilizing the HH in the glenoid fossa when the ISP malfunctions. Despite its relatively small moment arm and low clinical prominence in comparison with the SSP, ISP, and SSC, it may be useful in compensating for the concavity compression force [18,19]. Age-related changes in tendon quality, muscle elasticity, and neuromuscular control further modify these mechanisms, predisposing older patients to superior HH migration even in the presence of smaller or chronic tears. Furthermore, superior HH translation may be more prevalent in male patients, as they are more susceptible to RCTs than female patients [2].

Superior migration of the HH represents a final common pathway resulting from heterogeneous pathomechanical mechanisms, which may act independently or synergistically. Although superior migration of the HH is classically associated with RCTs, they are not its sole cause. This pathomechanism is also connected with some neuromuscular dysfunctions, osteoarthritis (OA), and variations in acromion morphology, especially type III [20-22]. The latter mechanism results in apparent elevation of the HH, as the morphology of the scapula is altered and subacromial impingement appears. According to Morelli et al., people with type III acromion have an almost threefold higher risk of RCT compared to those with type I or II acromion [22]. Neuromuscular impairments, such as suprascapular nerve dysfunction, cause SSP and ISP impairment through atrophy and loss of function [20,21]. Both of these phenomena weaken the RC, thus contributing to superior HH migration. Scapular dyskinesis also disrupts shoulder biomechanics, moving the HH away from the center of the GHJ, which can radiologically appear as superior HH migration [23,24]. Adhesive capsulitis is another shoulder dysfunction that may lead to superior HH migration. Contraction of the coracohumeral ligament, GHJ capsule, and rotator interval results in elevation of the HH - another mechanism unrelated to RCTs [25]. Rheumatoid arthritis is yet another cause of progressive GHJ destruction, along with superior migration of the HH. This is due to more rapid RC tendon degeneration and loss of their stabilizing function. Moreover, many authors found that it is more likely the severity of rheumatoid arthritis, rather than its duration, that causes these changes [18,26,27].

Diagnosis of HH elevation

Diagnosis of the superior migration of the HH may imply an insufficiency of the RC rather than a tear in a single, particular tendon. It may serve as a marker of the shoulder biomechanics disruption, especially chronic and massive RCTs. The diagnosis of the HH elevation comprises clinical function of the shoulder and diagnostic imaging, i.e., radiograms, ultrasound (US), and magnetic resonance imaging (MRI).

Although elevation of the HH can be relatively easily observed in anteroposterior radiograms as well as other diagnostic tools, it is worth starting the case with clinical tests commonly used for RCTs. It is important to notice that there is no specific test for superior migration of the HH, as it is a result of RC disease and not an independent problem. Thus, we should rely on the tests that are commonly used for RCTs. In the literature, recommended tests include the lift-off, belly press, and bear hug tests, which may indicate SSC tears; Jobe’s, Neer’s, drop arm, full can, and Hawkins-Kennedy tests for SSP tear detection; and the external rotation lag sign for ISP tears. The external rotation lag sign can be performed at 0° of abduction and at 90° of abduction [28,29]. Both variants have very high specificity (0.98 and 1, respectively), but low sensitivity (0.1 and 0.08, respectively). Thus, only a limited number of RCTs can be detected, but when the test is positive, one can be almost certain of an ISP tear.

A similar statement applies to the lift-off, belly press, and drop arm tests, as they also have low sensitivity and very high specificity for RCT detection [29]. There is incoherent data in the literature regarding the sensitivity and specificity of Jobe’s test, which is reported as 0.19-0.94 and 0.39-1, respectively. Jain et al., in their paper, hypothesize that this can be a result of subject heterogeneity and include only patients who underwent surgery in the research. Furthermore, the authors found that the full can test has a higher specificity and likelihood ratio than Jobe’s test; thus, it can be more useful in detecting SSP tears. For Neer’s and Hawkins-Kennedy tests, sensitivity is much higher than specificity - 0.6 and 0.35, respectively, for Neer’s test, and 0.77 and 0.26, respectively, for the Hawkins-Kennedy test. This implies that these tests are not highly indicative of a particular tendon tear but rather of a general shoulder issue, and the examiner can only suspect an RCT [29]. 

Radiologically, the most practical parameter is AHI, which is defined as the distance between the inferior border of the acromion and the uppermost part of the HH [6]. Its usefulness lies in its simplicity; it can be easily derived from a simple shoulder anteroposterior radiogram. It has been proven that elevation of the HH can reflect the severity of degeneration and indicate serious tendon retraction (Patte grade 3) and fatty infiltration (tangent sign, Goutallier grade >2). This further translates to worse prognosis, difficult arthroscopic repair, and a higher risk of progression to RCA [7,8,30-33]. AHI is often mentioned as a reliable tool in detecting these pathologies. Its normal value ranges from 4 to 17 mm, with a mean of 7.2-8.6 mm [6,8,31,33-35]. It is worth noting that AHI is significantly smaller for measurements done on MRI scans (6.4 mm) than in standard anteroposterior radiograms (7.2 mm) [8,31,33,35]. This may be caused by different patient positioning (supine), different muscle tension, or the better resolution and soft tissue imaging capabilities of MRI [33]. Although there is no proof of MRI studies’ superiority over radiograms in AHI measurement, the diagnostic value of MRI may be higher than that of a standard radiogram. AHI values <7 mm strongly suggest a massive RCT, whereas <6 mm is often linked with irreversible changes to the GHJ, a high risk of tendon repair failure, and RCA [6-8,30,33]. However, some studies suggest that AHI can be decreased below 7 mm with normal RC tendons and no clinical manifestations around the shoulder [8,33]. AHI is a cheap and simple marker of RC pathology, but it should not serve as the sole criterion for surgical qualification. It should be correlated with the clinical function of the RC and the MRI image of the shoulder.

The US is a fast, accessible, non-invasive, and radiation-free method of RC diagnostics, yet the outcome of the examination depends greatly on the abilities of the examiner [34,36,37]. However, nowadays its value is high, and it is performed in almost every orthopedic office. In a meta-analysis by Smith et al. [36], the authors found that US has an acceptable sensitivity and specificity, reaching 0.84 and 0.89, respectively, for partial tears, and 0.96 and 0.93, respectively, for full-thickness tears. Other studies also found that US has very high accuracy for partial and full-thickness RCTs [34,37]. This implies that US can be useful not only in detecting massive RCTs, but also in identifying early, small tears, which then allows for early treatment implementation.

Furthermore, the authors found that US examination is most reliable when performed by a musculoskeletal radiologist, followed by an orthopedic surgeon, and less reliable when performed by a general radiologist or ultrasonographer [36]. Overall, ultrasonography proves extremely helpful, but MRI offers a more global view of the shoulder, especially regarding deep structures such as the labrum, articular cartilage, and bone marrow. In addition, MRI is more useful in AHI measurement, as the subacromial space is inaccessible in US examination due to the acoustic shadow of the acromion [34,37].

Treatment of massive, irreparable rotator cuff tears (MIRCTs)

Superior HH migration, along with decreased AHI, can be used as a marker of clinical severity in RCTs. It correlates with worse shoulder function, worse tendon healing, and degenerative changes; thus, it can be perceived as a prognostic factor [7,8,30-33]. Early diagnosis of superior HH migration is crucial in order to stop the progression of the RCT to RCA and avoid irreversible changes to the shoulder. If possible - that is, depending on the radiological findings and the severity of changes - conservative treatment should be the first-line intervention. It is especially helpful in less advanced RCTs and functional disorders, such as scapular dyskinesis and adhesive capsulitis [38-40]. Conservative treatment is successful in up to 90% of patients with adhesive capsulitis and includes non-steroidal anti-inflammatory drugs (NSAIDs), physical therapy, exercise, corticosteroid injections, and hydrodissection, but it should be tailored to the phase of the disease [39,41]. A large multicenter study by Kuhn et al. has shown that appropriate conservative treatment can be successful in approximately 75% of cases of atraumatic, full-thickness RCTs. However, the authors state that they found superior HH migration in only 15% of patients; in 70%, the tear involved only the SSP tendon, and in most cases (81%), the retraction was Patte grade 1 or 2 [40]. Thus, the presented results refer mostly to low- or moderate-severity RCTs and cannot be translated to more advanced RCTs with superior HH migration. Nevertheless, nonoperative management should be attempted in MIRCTs because of the challenges in their surgical treatment and the potentially unnecessary nature of surgical intervention. Activation and strengthening of the anterior deltoid are especially beneficial, as it can compensate for dysfunctional RC muscles. On the other hand, prolonged conservative treatment can lead to progression of degenerative changes and complicate future surgical management if needed [42,43].

RC irreparability factors have been universally determined in the literature as advanced superior humeral migration with AHI reduction, massive tears, severe fatty infiltration, and muscle atrophy [43-47]. When nonoperative treatment fails, or there is substantial pain, surgery is indicated. Nowadays, there is a wide variety of procedures that can be proposed to a patient with MIRCT. They include arthroscopic debridement, partial repair, superior capsular reconstruction (SCR), graft interposition, balloon spacer arthroplasty, trapezius transfer (TT), latissimus dorsi transfer (LDT), and finally, reverse shoulder arthroplasty (RSA) [15,42,43,46,47]. The preferred method depends mostly on the patient profile - that is, his age and expectations regarding a return to prior activity. Generally, in older patients with low demands on weight bearing and activity, RSA is the preferred intervention, whereas younger patients who want to remain active benefit more from musculotendinous transfers [46]. 

In MIRCTs, a partial repair may be a reliable option, aiming to repair as much of the tendon as possible and restore shoulder biomechanics. Even nonanatomic, incomplete repair can improve function, decrease pain, and reduce superior HH migration. It is typically indicated in patients with irreparable SSP, repairable ISP and SSC, who do not have glenohumeral arthritis but have persistent pain despite well-performed conservative treatment [43]. However, this method has limited effectiveness in long-term follow-up and a high retear rate, reaching as much as 41.6% according to Chen et al. The authors found that AHI is not a relevant indicator of surgical outcome in this technique, nor are age, sex, diabetes status, smoking status, or preoperative duration of symptoms. They reported that a lower preoperative ASES score, a higher preoperative VAS score, and night pain were associated with greater functional improvement [48].

Cuff et al. demonstrated that in patients with MIRCTs and preserved overhead motion, partial repair along with biceps tenotomy is perceived as satisfying by 75% of patients after a five-year follow-up and fails in 26%. They found that patients had improved ASES scores (46.6 to 79.3 points; p < 0.001) and decreased VAS pain scores (6.9 to 1.9; p < 0.001). However, no improvement in range of motion was observed, and 36% of patients had progression of HH elevation. The authors highlight that this method requires revision procedures, such as RSA, in some cases but “does not burn bridges” for further intervention [49].

In recent years, SCR has gained increasing attention as a method that aims to restore the superior GHJ capsule and stabilize the HH. It was proposed as a treatment option for MIRCTs by Mihata et al. in 2012, useful in patients with irreparable RCTs, superior HH migration, and subjective shoulder function loss without significant OA [15,43,47]. This procedure utilizes a fascia lata autograft, which is arthroscopically attached medially to the superior glenoid and laterally to the greater tuberosity, with simultaneous stitches connecting the graft to the remaining RC tendons. There are also reports describing the use of acellular dermal allograft, LHBT (long head of the biceps tendon) autograft or rerouting, and semitendinosus tendon autograft or allograft for SCR [43,47].

A following paper by Mihata et al. investigated the effectiveness of SCR. The authors found significant improvements in the ASES score (23.5 to 92.9 points; p < 0.0001) and UCLA score (9.9 to 32.4 points; p < 0.00001). Moreover, average active elevation and external rotation both increased significantly by 64° (p < 0.001) and 14° (p < 0.01), respectively. The AHI improved from 4.6 mm to 8.7 mm (p < 0.001), and no progression of OA or RC muscle atrophy occurred in any patient. The average follow-up was 34.1 months [50].

Other authors found similar improvements in shoulder assessment scores, shoulder function, and AHI, supporting the usefulness of SCR and its efficacy, but they also reported a failure rate of 25.4% to 36.1% [51-53]. Lee and Min found that a small improvement in AHI immediately postoperatively, along with loss of the integrity of the posterior remnant tissue, was associated with retears and could be used as a predictive factor for SCR success [53]. According to Mihata et al., shoulders with a postoperative AHI of 5 mm or less had experienced postoperative retears of the repaired ISP tendon or graft tear, and the overall failure rate was 16.7% [50]. Nonetheless, the improvement of AHI is uncertain at long-term follow-up, and further studies should be performed.

Nowadays, musculotendinous transfers involve mainly lower trapezius transfer (LTT) and LDT due to their superior biomechanical properties and greater effectiveness compared with pectoralis major, pectoralis minor, or teres major transfers. Historically, pectoralis major has been the most popular procedure of this type, but it fails to restore shoulder strength and function at the demanded level. LTT and LDT are procedures that can be implemented as reconstruction techniques in postero-superior RCTs due to their similar pull force direction [43,46]. Although primarily performed as an open procedure, Elhassan et al. in 2016 reported arthroscopically assisted LTT. It included detachment of the lower trapezius from the scapular spine and reconstruction of the SSP with an Achilles tendon allograft attached to the SSP footprint. This type of procedure is indicated in young patients with MIRCTs and minimal to mild GHJ OA [54]. Another study by Elhassan et al. described the outcomes of LTT and found significant improvement in shoulder function and range of motion. AHI improved from 2.3 to 8 mm, and the failure rate was 3%. Furthermore, they concluded that a better preoperative range of motion was an indicator of superior postoperative outcomes [55]. LTT has a better biomechanical outcome in external rotation restoration than LDT and should be the preferred technique [56].

RSA is a salvage procedure, reasonable when RCA has developed or after failure of other surgical methods. It is primarily indicated for older, low-demand patients, though, according to newer studies, younger patients also benefit from RSA [4,57]. In this procedure, the HH is medialized and distalized, which gives better biomechanical properties and provides better deltoid function, compensating for impaired RC muscles [4]. Recent advancements in the RSA technique support better outcomes regarding range of motion, little to no pain, and high patient-reported satisfaction postoperatively. However, the procedure is technically demanding, and the reported complication rate is relatively high, reaching up to 71% in some series. Complications include aseptic loosening, instability, implant dissociation, infection, fracture, neurapraxia, and radiographic signs of scapular notching. As many as 26% of patients need revision [3]. Furthermore, it was found that unsuccessful RCT repair prior to RSA was associated with worse outcomes regarding range of motion, pain, and functional outcome scores than primary RSA [58]. Thus, patients with a high risk of retear after RCT repair - especially in the presence of failure markers - should rather be treated with primary RSA. Superior migration of the HH and decreased AHI may serve as such markers. Table 2 provides a summary of treatment strategies for RCTs in selected clinical scenarios, considering key decision-making factors.

**Table 2:** Summary of treatment strategies for RCTs in selected clinical scenarios, considering key decision-making factors (age, glenohumeral osteoarthritis severity, acromiohumeral interval/superior humeral head migration, and patient activity level). RCTs, rotator cuff tears; AHI, acromiohumeral interval; HH, humeral head; NSAIDs, non-steroidal anti-inflammatory drugs; MIRCT, massive, irreparable rotator cuff tear; SSP, supraspinatus; ISP, infraspinatus; SSC, subscapularis; SCR, superior capsular reconstruction; RSA, reverse shoulder arthroplasty; RCA, rotator cuff arthropathy

Clinical scenario	Age	Glenohumeral osteoarthritis severity	AHI/HH migration	Patient activity level	Recommended treatment
Early/low-grade RCT	Any	None	Preserved AHI, no superior HH migration	Any	Conservative treatment (NSAIDs, physiotherapy, exercises, injections) [38-40]
RCT with functional disorders (scapular dyskinesis, adhesive capsulitis)	Any	None	Preserved AHI	Any	Tailored conservative treatment, phase-dependent [38-41]
Atraumatic full-thickness RCT, low-moderate severity	Any	None-mild	Minimal HH migration, preserved AHI	Any	Initial nonoperative management [40]
Massive RCT without superior HH migration	<65	None-mild	Mild AHI reduction	Moderate-high	Attempt conservative treatment, monitor progression [40,42,43]
MIRCT (irreparable SSP, repairable ISP/SSC), no osteoarthritis	<65	None	Reduced AHI, superior HH migration	Moderate-high	Partial repair ± biceps tenotomy [43,48,49]
MIRCT with superior HH migration, preserved joint	<65	None-mild	Markedly reduced AHI	High	SCR [15,43,47,50-53]
MIRCT, posterosuperior tear pattern	<65	None-mild	Reduced AHI	High	Musculotendinous transfer (preferably lower trapezius transfer) [43,46,54-56]
MIRCT with failed conservative treatment	≥65	Mild-moderate	Reduced AHI	Low	RSA or limited surgical options [42,46,57]
RCA	≥65	Advanced	Severe HH migration, collapsed AHI	Low	RSA [3,4,57]
High risk of retear (very low AHI, marked HH migration)	Any	Any	Severely reduced AHI	Any	Prefer primary RSA over cuff repair [43,58]
Failed prior RCT repair	Any	Any	Persistent HH migration	Any	RSA preferred over revision repair [58]

It is important to highlight some potential limitations of this study. This study is a narrative review and does not adhere to the rigorous methodological standards of a systematic review. Therefore, some degree of subjectivity in the selection and interpretation of the literature cannot be excluded. Furthermore, no new statistical analyses were performed, and this review relies on data reported in the original source studies. The available literature on chronic RCTs is extensive, yet heterogeneous, and only a limited number of studies provide clear recommendations regarding massive RCT treatment. We encourage further high-quality research aimed at developing a standardized treatment algorithm.

## Conclusions

There is currently a limited number of studies that define a clear treatment strategy based on a specific stage of superior HH migration. Decreased AHI correlates with RCT severity, fatty infiltration grade, and retear rates. Although AHI is a simple, inexpensive, and easily accessible marker of RC pathology, it should not serve as a sole criterion for surgical decision-making. Treatment should be individualized depending on the patient's age, activity demands, and glenohumeral OA severity. SCR, partial repair, and LTTs are associated with improved AHI and shoulder function, but in cases with advanced RC arthropathy, RSA is the most reliable option.

## References

[1] Kolk A, Henseler JF, de Witte PB (2017). The effect of a rotator cuff tear and its size on three-dimensional shoulder motion. Clin Biomech (Bristol).

[2] Yamamoto A, Takagishi K, Osawa T, Yanagawa T, Nakajima D, Shitara H, Kobayashi T (2010). Prevalence and risk factors of a rotator cuff tear in the general population. J Shoulder Elbow Surg.

[3] Nam D, Maak TG, Raphael BS, Kepler CK, Cross MB, Warren RF (2012). Rotator cuff tear arthropathy: evaluation, diagnosis, and treatment: AAOS exhibit selection. J Bone Joint Surg Am.

[4] Clifford AL, Hurley E, Anakwenze O, Klifto CS (2024). Rotator cuff arthropathy: A comprehensive review. J Hand Surg Glob Online.

[5] Chen C, Wu C, Xu J (2022). Are scapular morphologic characteristics or rotator cuff tear patterns associated with acetabularization of the coracoacromial arch?. JSES Int.

[6] Weiner DS, Macnab I (1970). Superior migration of the humeral head. A radiological aid in the diagnosis of tears of the rotator cuff. J Bone Joint Surg Br.

[7] Razmjou H, Palinkas V, Christakis M, Kennedy D, Robarts S (2020). Diagnostic value of acromiohumeral distance in rotator cuff pathology: implications for advanced-practice physiotherapists. Physiother Can.

[8] Boyle AB, MacLean SB (2025). Redefining superior escape of the humeral head: a radiographic and magnetic resonance imaging study. Shoulder Elbow.

[9] Walch G, Edwards TB, Boulahia A, Nové-Josserand L, Neyton L, Szabo I (2005). Arthroscopic tenotomy of the long head of the biceps in the treatment of rotator cuff tears: clinical and radiographic results of 307 cases. J Shoulder Elbow Surg.

[10] Hamada K, Yamanaka K, Uchiyama Y, Mikasa T, Mikasa M (2011). A radiographic classification of massive rotator cuff tear arthritis. Clin Orthop Relat Res.

[11] Akhtar A, Richards J, Monga P (2021). The biomechanics of the rotator cuff in health and disease - a narrative review. J Clin Orthop Trauma.

[12] Yamamoto N, Itoi E (2015). A review of biomechanics of the shoulder and biomechanical concepts of rotator cuff repair. Asia Pac J Sports Med Arthrosc Rehabil Technol.

[13] Longo UG, Berton A, Papapietro N, Maffulli N, Denaro V (2012). Biomechanics of the rotator cuff: European perspective. Med Sport Sci.

[14] Lippitt SB, Vanderhooft JE, Harris SL, Sidles JA, Harryman DT, Matsen FA (1993). Glenohumeral stability from concavity-compression: a quantitative analysis. J Shoulder Elbow Surg.

[15] Mihata T, McGarry MH, Pirolo JM, Kinoshita M, Lee TQ (2012). Superior capsule reconstruction to restore superior stability in irreparable rotator cuff tears: a biomechanical cadaveric study. Am J Sports Med.

[16] Steenbrink F, de Groot JH, Veeger HE, van der Helm FC, Rozing PM (2009). Glenohumeral stability in simulated rotator cuff tears. J Biomech.

[17] Magarey ME, Jones MA (2003). Specific evaluation of the function of force couples relevant for stabilization of the glenohumeral joint. Man Ther.

[18] van de Sande MA, de Groot JH, Rozing PM (2008). Clinical implications of rotator cuff degeneration in the rheumatic shoulder. Arthritis Rheum.

[19] de Groot JH, van de Sande MA, Meskers CG, Rozing PM (2006). Pathological teres major activation in patients with massive rotator cuff tears alters with pain relief and/or salvage surgery transfer. Clin Biomech (Bristol).

[20] Werner CM, Weishaupt D, Blumenthal S, Curt A, Favre P, Gerber C (2006). Effect of experimental suprascapular nerve block on active glenohumeral translations in vivo. J Orthop Res.

[21] Bozzi F, Alabau-Rodriguez S, Barrera-Ochoa S, Ateschrang A, Schreiner AJ, Monllau JC, Perelli S (2020). Suprascapular neuropathy around the shoulder: a current concept review. J Clin Med.

[22] Morelli KM, Martin BR, Charakla FH, Durmisevic A, Warren GL (2019). Acromion morphology and prevalence of rotator cuff tear: a systematic review and meta-analysis. Clin Anat.

[23] Kibler WB, Sciascia A (2010). Current concepts: scapular dyskinesis. Br J Sports Med.

[24] Deutsch A, Altchek DW, Schwartz E, Otis JC, Warren RF (1996). Radiologic measurement of superior displacement of the humeral head in the impingement syndrome. J Shoulder Elbow Surg.

[25] Dimitriou D, Mazel P, Hochreiter B, Fritz B, Bouaicha S, Wieser K, Grubhofer F (2021). Superior humeral head migration might be a radiological aid in diagnosing patients with adhesive capsulitis of the shoulder. JSES Int.

[26] Hirooka A, Wakitani S, Yoneda M, Ochi T (1996). Shoulder destruction in rheumatoid arthritis. Classification and prognostic signs in 83 patients followed 5-23 years. Acta Orthop Scand.

[27] Kelly IG (1994). Unconstrained shoulder arthroplasty in rheumatoid arthritis. Clin Orthop Relat Res.

[28] Phillips N (2014). Tests for diagnosing subacromial impingement syndrome and rotator cuff disease. Shoulder Elbow.

[29] Jain NB, Luz J, Higgins LD, Dong Y, Warner JJ, Matzkin E, Katz JN (2017). The diagnostic accuracy of special tests for rotator cuff tear: the ROW cohort study. Am J Phys Med Rehabil.

[30] Nové-Josserand L, Edwards TB, O'Connor DP, Walch G (2005). The acromiohumeral and coracohumeral intervals are abnormal in rotator cuff tears with muscular fatty degeneration. Clin Orthop Relat Res.

[31] Park SH, Choi CH, Yoon HK, Ha JW, Lee C, Chung K (2020). What can the radiological parameters of superior migration of the humeral head tell us about the reparability of massive rotator cuff tears?. PLoS One.

[32] Werner BS, Jacquot A, Molé D, Walch G (2016). Is radiographic measurement of acromiohumeral distance on anteroposterior view after reverse shoulder arthroplasty reliable?. J Shoulder Elbow Surg.

[33] Saupe N, Pfirrmann CW, Schmid MR, Jost B, Werner CM, Zanetti M (2006). Association between rotator cuff abnormalities and reduced acromiohumeral distance. AJR Am J Roentgenol.

[34] McCreesh KM, Crotty JM, Lewis JS (2015). Acromiohumeral distance measurement in rotator cuff tendinopathy: is there a reliable, clinically applicable method? A systematic review. Br J Sports Med.

[35] Werner CM, Conrad SJ, Meyer DC, Keller A, Hodler J, Gerber C (2008). Intermethod agreement and interobserver correlation of radiologic acromiohumeral distance measurements. J Shoulder Elbow Surg.

[36] Smith TO, Back T, Toms AP, Hing CB (2011). Diagnostic accuracy of ultrasound for rotator cuff tears in adults: a systematic review and meta-analysis. Clin Radiol.

[37] Levine BD, Motamedi K, Seeger LL (2012). Imaging of the shoulder: a comparison of MRI and ultrasound. Curr Sports Med Rep.

[38] Longo UG, Risi Ambrogioni L, Berton A (2020). Scapular dyskinesis: from basic science to ultimate treatment. Int J Environ Res Public Health.

[39] Cho CH, Bae KC, Kim DH (2019). Treatment strategy for frozen shoulder. Clin Orthop Surg.

[40] Kuhn JE, Dunn WR, Sanders R (2013). Effectiveness of physical therapy in treating atraumatic full-thickness rotator cuff tears: a multicenter prospective cohort study. J Shoulder Elbow Surg.

[41] Levine WN, Kashyap CP, Bak SF, Ahmad CS, Blaine TA, Bigliani LU (2007). Nonoperative management of idiopathic adhesive capsulitis. J Shoulder Elbow Surg.

[42] Neri BR, Chan KW, Kwon YW (2009). Management of massive and irreparable rotator cuff tears. J Shoulder Elbow Surg.

[43] Carver TJ, Kraeutler MJ, Smith JR, Bravman JT, McCarty EC (2018). Nonarthroplasty surgical treatment options for massive, irreparable rotator cuff tears. Orthop J Sports Med.

[44] Hsu KL, Kuan FC, Velasquez Garcia A, Hong CK, Chen Y, Shih CA, Su WR (2024). Factors associated with reparability of rotator cuff tears: a systematic review and meta-analysis. J Shoulder Elbow Surg.

[45] Kim IB, Jung DW, Suh KT (2018). Prediction of the irreparability of rotator cuff tears. Arthroscopy.

[46] Tytgat H, Macdonald P, Verhaegen F (2024). Management of irreparable subscapularis tears: current concepts. J ISAKOS.

[47] Dey Hazra ME, Dey Hazra RO, Hanson JA (2023). Treatment options for massive irreparable rotator cuff tears: a review of arthroscopic surgical options. EFORT Open Rev.

[48] Chen KH, Chiang ER, Wang HY, Ma HL (2017). Arthroscopic partial repair of irreparable rotator cuff tears: factors related to greater degree of clinical improvement at 2 years of follow-up. Arthroscopy.

[49] Cuff DJ, Pupello DR, Santoni BG (2016). Partial rotator cuff repair and biceps tenotomy for the treatment of patients with massive cuff tears and retained overhead elevation: midterm outcomes with a minimum 5 years of follow-up. J Shoulder Elbow Surg.

[50] Mihata T, Lee TQ, Watanabe C, Fukunishi K, Ohue M, Tsujimura T, Kinoshita M (2013). Clinical results of arthroscopic superior capsule reconstruction for irreparable rotator cuff tears. Arthroscopy.

[51] Pennington WT, Bartz BA, Pauli JM, Walker CE, Schmidt W (2018). Arthroscopic superior capsular reconstruction with acellular dermal allograft for the treatment of massive irreparable rotator cuff tears: short-term clinical outcomes and the radiographic parameter of superior capsular distance. Arthroscopy.

[52] Denard PJ, Brady PC, Adams CR, Tokish JM, Burkhart SS (2018). Preliminary results of arthroscopic superior capsule reconstruction with dermal allograft. Arthroscopy.

[53] Lee SJ, Min YK (2018). Can inadequate acromiohumeral distance improvement and poor posterior remnant tissue be the predictive factors of re-tear? Preliminary outcomes of arthroscopic superior capsular reconstruction. Knee Surg Sports Traumatol Arthrosc.

[54] Elhassan BT, Alentorn-Geli E, Assenmacher AT, Wagner ER (2016). Arthroscopic-assisted lower trapezius tendon transfer for massive irreparable posterior-superior rotator cuff tears: surgical technique. Arthrosc Tech.

[55] Elhassan BT, Wagner ER, Werthel JD (2016). Outcome of lower trapezius transfer to reconstruct massive irreparable posterior-superior rotator cuff tear. J Shoulder Elbow Surg.

[56] Hartzler RU, Barlow JD, An KN, Elhassan BT (2012). Biomechanical effectiveness of different types of tendon transfers to the shoulder for external rotation. J Shoulder Elbow Surg.

[57] Thorsness R, Romeo A (2016). Massive rotator cuff tears: trends in surgical management. Orthopedics.

[58] Welch JM, Hurley ET, Lorentz S (2024). Reverse shoulder arthroplasty following failed rotator cuff repair: a systematic review and meta-analysis. Shoulder Elbow.

